# Metacognitive sensitivity and symptoms of mental disorder: A systematic review and meta-analysis

**DOI:** 10.3389/fpsyg.2023.991339

**Published:** 2023-02-02

**Authors:** Marianne Hohendorf, Markus Bauer

**Affiliations:** School of Psychology, University of Nottingham, Nottingham, United Kingdom

**Keywords:** metacognition, mental disorder, psychiatric, metacognitive sensitivity, experimental, decision making, systematic review and meta-analysis

## Abstract

**Introduction:**

Metacognition is a term used to refer to cognition about cognitive processes. In this systematic review and meta-analysis, we reviewed studies that investigated the relationship between experimentally measured objective metacognitive sensitivity and diverse symptoms of mental disorder. In these studies, metacognitive sensitivity is operationalized as the correspondence between the accuracy of task performance and reported confidence therein.

**Methods:**

A literature search was conducted across four databases and studies were selected for review based on predefined eligibility criteria. Twenty studies were included in the review and separate meta-analyses were conducted for psychotic and non-psychotic categories of psychiatric symptoms.

**Results:**

A significant reduction (medium effect size) in metacognitive sensitivity was found in individuals with psychosis-related symptoms of mental disorder compared to healthy control groups, but no significant difference was found for individuals with non-psychotic symptoms. It should be noted though, that fewer studies were available for the latter group. Sub-group analysis found no evidence that the effect of metacognitive impairment depended on whether perceptual or non-perceptual experimental tasks were employed.

**Discussion:**

These findings are discussed in relation to other conceptualizations of metacognition and the role reduced metacognitive sensitivity may play in forms of mental disorder.

## 1. Introduction

Cognition can be described as the mental processes through which “sensory input is transformed, reduced, elaborated, stored, recovered, and used” (Neisser, 1967). Mental processes that are considered to be “cognition about cognitive phenomena” (Flavell, 1979, p. 906) rather than sensory input have been termed ‘metacognition’. Metacognition is usually distinguished from the operations of executive function, which also have other cognitive processes as their object, in that the former is considered to require conscious representation of the cognitive process in question (Heyes et al., 2020). Forms of metacognition have been differentiated based on the kinds of cognitive processes involved. The cognitive phenomena that are the object of metacognition, referred to as “first-order cognition,” can be assigned to different cognitive domains such as memory and perception, and are employed in monitoring and regulating interaction with stimuli. Cognition about these first-order processes, i.e., metacognition or “second-order cognition,” monitors and regulates the represented first-order processes. Performance in tasks requiring second-order cognition, referred to as “type-2” tasks (Clarke et al., 1959), has been found to be dissociable from that in tasks based only on first-order cognition, referred to as “type-1” tasks (e.g., Fleming et al., 2010; Rouault et al., 2018b). Where monitoring of first-order cognition produces a representation of the cognitive process that closely reflects its relation to environmental input, this can be considered high metacognitive sensitivity or accuracy. Whether the representation is of one’s own cognitive processes or those of others, accurate metacognition promotes adaptive behavior at an individual and interpersonal level (David et al., 2012) and has thus been an avenue for developing treatments of mental disorder (Moritz and Lysaker, 2018).

Metacognition can be considered at either the local or global level (Seow et al., 2021). The global level of metacognition pertains to how an individual monitors and regulates patterns of mental processes that can be considered general properties of themselves or of others across contexts, an example of which may be an individual’s perception of their ability to recognize previously encountered faces compared to their ability to recognize objects. On the other hand, local metacognition refers to an individual’s monitoring and regulation of their own mental activity where it is integral to the performance of a discrete task, such as the extent to which their recognition of a particular object is a reliable indication that they previously encountered that object. The two levels of metacognition are measured using different approaches. A profile of global metacognition is typically produced by asking individuals to report beliefs regarding their mental processes generally or conducting interviews to infer these attributions (e.g., Morrison et al., 2007; Sellers et al., 2017), whereas local metacognition is measured by comparing individuals’ first-order cognition, assessed objectively through behavioral tasks, to their self-reported perceptions of performance within these tasks.

Both local and global metacognition have been assessed alongside indicators of mental health in clinical (Davies et al., 2018) and non-clinical populations (Chan et al., 2015), as well as using transdiagnostic approaches (Rouault et al., 2018b). Research in the latter case has occurred as part of a movement toward measuring symptom dimensions to overcome problems arising from comorbidity and symptom variability within diagnostic categories (Gillan et al., 2016; Seow et al., 2021). Both local and global manifestations of metacognition have been found to be associated with symptoms of mental disorder (Cooper and Osman, 2007; Bliksted et al., 2017) as well as clinical insight, which depends on accurate assessment of cognitive functions where these are related to clinical diagnosis. For this reason, metacognition has been a target for change in treatment settings through various programs (Van Oosterhout et al., 2016; Moritz and Lysaker, 2018). These programmes, which promote adaptive ways of relating to mental experience, have been tailored more to global metacognition than local metacognition (Lysaker et al., 2018). Seow et al. (2021) however, argue that there is likely a bidirectional influence between the two levels of metacognition, given evidence that global metacognition is likely to influence how an individual monitors and regulates their task-specific cognition (Rouault and Fleming, 2020), while metacognition employed with respect to a particular task shapes a person’s global self-performance attributions (Rouault et al., 2019; Lee et al., 2021). Local metacognition (hereafter “metacognition” unless otherwise specified) measured experimentally combines behavioral measures from a standardized task and self-report measures relating to perceived performance. This operationalization of metacognition provides a standardized index of first-order cognitive processes for comparison with and evaluation of self-reported perceptions of cognition.

Metacognition is most often gauged by eliciting post-response statements of confidence in task performance. Using this experimental operationalization, metacognitive monitoring is the relationship between confidence and response accuracy, where adaptive metacognition is demonstrated through high confidence reports after accurate task performance and low confidence after inaccurate task performance. Metacognitive evaluation expressed as confidence with regards to response accuracy is typically considered to be influenced by two distinct properties (Maniscalco and Lau, 2012), which are bias and sensitivity. This review will focus on metacognitive sensitivity, or the extent to which an individual’s confidence discriminates their first-order accuracy. Some researchers have inferred metacognitive sensitivity simply through the strength of correlation between confidence and decision accuracy (Nelson et al., 1986), while others have used metrics derived from signal detection theory (SDT) to index “type-2” sensitivity (Galvin et al., 2003; Masson and Rotello, 2009) separately from response bias.

In tasks of first-order cognition, SDT is taken to measure response sensitivity, type-1 d’, independently from response bias where two external stimulus alternatives exist by comparing responses indicative of a stimulus when it is present, a participant’s “hits,” to such responses when the stimulus is absent, a participant’s “false alarms.” This analysis has been extended to measure metacognitive or “type-2” sensitivity by considering “hits” as responses of high confidence where accurate first-order performance is present in the form of type-1 hits or correct rejections, and “false alarms” where high confidence responses follow inaccurate first-order performance in the form of type-1 misses or false alarms. Type-1 and type-2 responses characterized according to SDT are described in Tables 1, 2, respectively.

**TABLE 1:** Type-1 SDT categories for signal responses as related to signal presence, * and ** denote the correct and incorrect type-1 responses.

	Signal-present response	Signal-absent response
Signal present	Hit*	Miss **
Signal absent	False alarm **	Correct rejection*

See Fleming and Dolan (2012).

**TABLE 2:** Type-2 SDT categories for confidence responses and their relation to type-1 accuracy.

	High confidence	Low confidence
Correct type-1 response (*)	Type-2 hit	Type-2 miss
Incorrect type-1 response (**)	Type-2 false alarm	Type-2 correct rejection

A correct type-1 response that is judged as being given with high confidence would be a type-2 hit, whereas an incorrect type-1 response that is met with high confidence is a type-2 false alarm. See Fleming and Dolan (2012). * and ** denote the correct and incorrect type-1 responses.

As confidence responses, unlike type-1 responses, are not considered to fulfill SDT assumptions of Gaussian distribution (Galvin et al., 2003), researchers have implemented SDT analysis of type-2 responses by calculating Receiver Operating Characteristic (ROC) curves based on hit and false alarm rates for individual confidence criteria, where the area under a type-2 ROC curve can be interpreted as a measure of metacognitive sensitivity.

**FIGURE 1:**
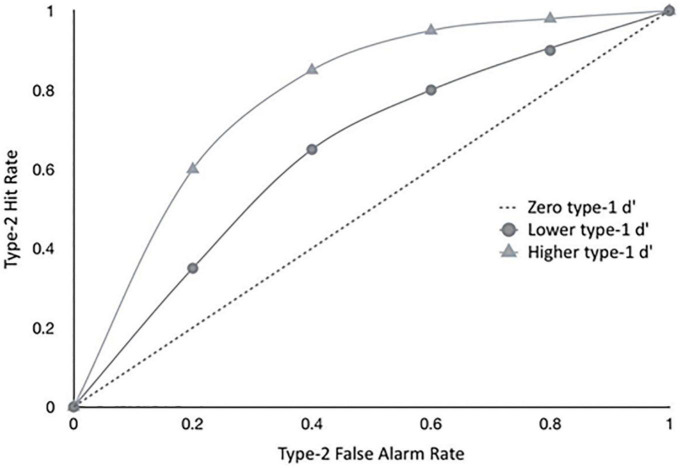
The graph shows hypothetical type II ROC curves of a hypothetical observer with a given metacognitive capacity, exposed to different levels of difficulty in the primary task, implying different levels of type I sensitivity (d’). Empirical metacognitive sensitivity (type II) will be constrained by type I task difficulty - the same observer will show different levels of type II sensitivities, depending on the difficult in the primary task. This is why we only considered studies that had approximately equal type I performance levels for both participant groups. See also Fleming and Dolan (2012).

Type-2 ROC measures are however influenced by type-1 performance (Galvin et al., 2003; Evans and Azzopardi, 2007), as illustrated in Figure 1, which shows theoretical ROC curves for the same individual for different levels of type-1 d’. To avoid confounding metacognitive sensitivity with first-order accuracy, a model-based SDT metric has been developed more recently which takes type-1 performance into account for estimates of metacognitive sensitivity (Maniscalco and Lau, 2012).

The influence of subjective task difficulty on measures of metacognitive sensitivity can be intuitively understood by considering the following example. In a visual detection task, a person would practically always be able to say whether they made an error in indicating the presence of a stimulus, i.e., that they have low confidence in their incorrect response, when that stimulus is highly visible to them in every “stimulus-present” trial, which would suggest perfect metacognitive sensitivity. However, the same individual would less reliably match their confidence to their accuracy in the same task with a less visible stimulus. This reduction in estimates of metacognitive sensitivity for difficult tasks is undesirable when attempting to assess an individual’s metacognitive sensitivity, which may be considered to be a stable property with respect to any task involving the same first order processes (Fleming et al., 2010). Where research attempts to assess metacognitive sensitivity in populations with different levels of psychiatric symptoms, which are likely to also differ in their first-order cognitive performance (Chan, 2016; Davies and Greenwood, 2020), it is particularly relevant to control for the latter either through experimental design or response analysis. A selection of the measures that have been used in estimating metacognitive sensitivity are summarized in Table 3.

**TABLE 3:** Measures used to represent metacognitive sensitivity from accuracy and confidence reports.

Measure	Description	Greater metacognitive sensitivity indicated by:	Limitations
Low confident incorrect responses	Number of incorrect responses with low confidence	Higher value	Influenced by first-order performance and metacognitive bias
High confident incorrect responses	Number of incorrect responses with high confidence	Lower value	Influenced by first-order performance and metacognitive bias
Goodman-Kruskall gamma coefficient Goodman and Kruskal (1954)	Correlation between confidence and accuracy	Higher value	Influenced by first-order performance and metacognitive bias
Confidence gap Moritz et al. (2003)	Mean confidence for correct responses - mean confidence for incorrect response	Higher value	Influenced by first-order performance
Knowledge Corruption Index Moritz et al. (2004)	Proportion of high confidence responses given for incorrect trials compared to total number of high confidence responses	Lower value	Influenced by first- order performance
AUROC2 Galvin et al. (2003)	Area under the Receiver Operating Characteristic (ROC) curve for type-2 responses.	Higher value	Influenced by first- order performance
Meta-*d’*/*d’* or Meta-*d’*-*d’* Rounis et al. (2010), Maniscalco and Lau (2012)	Type-1 sensitivity expected for metacognitively ideal individual, calculated from observed secondorder responses, compared to observed type-1 sensitivity.	Higher value	Requires extensive data or hierarchical Bayesian models for reliable interpretation

The limitations of these measures have been discussed by Fleming (2017) and Hoven et al. (2019).

Given that second-order cognition may be dissociated from first-order cognition (Fleming et al., 2010; De Gardelle and Mamassian, 2015; Desender et al., 2016), this suggests that metacognition does not depend only on the information from internal responses involved in first-order processes, but also on further cues such as the subjective experience of fluency or speed of the preceding decision (Fleming and Dolan, 2012). Since different processes are considered to contribute to first-order and metacognitive responses, it is possible that in some cases of mental disorder the cognitive processes for first-order responses are impaired while those for metacognitive ones are spared (Powers et al., 2017; Faivre et al., 2021), or vice versa (Chan et al., 2015; Berna et al., 2019). This dissociation also provides that an individual’s metacognitive sensitivity may be based on similar second-order processing across tasks from different first-order cognitive domains such as memory and perception. Some research findings have promoted a domain-specific conceptualization of metacognition insofar as it can be enhanced or impaired in one domain but not another (Baird et al., 2014; Fleming et al., 2014), which is supported by evidence of domain-specific patterns of activity in the prefrontal cortex as predicting metacognition, alongside a wider network of domain-general signals (Morales et al., 2018). However, reviews have reported inconclusive findings on the domain-specificity of metacognition (Rouault et al., 2018a; Vaccaro and Fleming, 2018).

Understanding the contribution of metacognition to symptoms of mental disorder depends on evaluating whether metacognitive sensitivity is specific to cognitive domain and whether variation exists at the level of local metacognitions, as opposed to their synthesis into global metacognitions (Seow et al., 2021). These insights will help to clarify at which level of metacognition treatments for psychopathology may function (Moritz and Lysaker, 2018). This systematic review and meta-analysis aims to evaluate whether local metacognitive monitoring varies in those with symptoms of mental disorder compared to those without such symptoms, and if so whether this relationship is found across cognitive domains.

## 2. Materials and methods

This systematic review and its meta-analyses have been conducted in line with PRISMA recommendations (Moher et al., 2009), in order to promote the replicability of findings and to increase confidence that conclusions regarding the research question are not based on a biased sample of available evidence. A protocol for the review was not registered but all other PRISMA procedural recommendations were followed as closely as possible and are described below.

### 2.1. Eligibility criteria

The eligibility of a study for inclusion in the review was determined according to the following pre-defined criteria. To be included, studies had to provide data on participants’ response accuracy in a behavioral task and its relationship to explicit reports of response confidence on a rating scale for after each trial. To reduce the possibility of bias in estimates of metacognition as discussed by Fleming and Lau (2014), studies were only included if performance on the behavioral task was equated or was shown to be not significantly different between groups. In addition, studies needed to have compared metacognition in participants grouped according to the presence and absence of psychiatric symptoms, i.e., in a binary fashion, rather than a continuous correlation between metacognitive sensitivity and symptom severity in a healthy population.

Studies that measured mental disorder or atypicality predominantly attributed to neurodevelopmental processes, neurodegenerative processes, neurological symptoms or brain injury and which can be dissociated from subjective mental well-being were excluded. Studies not published as English-language articles in peer-reviewed journals were excluded from this review.

### 2.2. Study search and selection strategy

Records of studies were sourced through searches of the online databases APA PsychInfo, PubMed, Scopus and Web of Science during October 2022. Further potentially relevant studies were then sourced by manually searching relevant review publications returned by the database searches, as well as citations from database-retrieved studies which had been identified as meeting the pre-defined inclusion criteria after full-text screening.

The choice of search terms was partly guided by those used by Hoven et al. (2019), who examined confidence in relation to psychopathology, with the addition of terms excluding studies based on questionnaire measures of attributive metacognition. The databases were searched for articles whose titles included any of the terms: “metacogniti*” or “metamemory” and either “psychiatr*” or “impulsiv*” or “compulsiv*” or “symptom*” or “depressi*” or “schizo*” or “OCD” or “addict*” or “substance*” or “eating” or “MDD” or “gambl*” or “anxi*” or “psychos*” or “disorder*,” but without “treatment*”, “training,” “therap*” or “belief*” in the abstract or title.

Duplicates were removed from the collection of article records produced by combining the four database searches and then the titles and abstracts of the records were screened in order to assess whether they could meet the pre-established inclusion criteria. Where it seemed possible that a study met inclusion criteria based on the title and abstract, the full text was retrieved and screened to confirm eligibility for inclusion. The reference sections of articles confirmed as eligible for inclusion in the review were searched for further potentially relevant articles. Where data necessary for effect size calculation was not available in the published article, the corresponding author named in the article was contacted with a request for the information required to calculate the effect size. For studies where no data was made available (or would have required substantial re-analyses) to the authors of this review, effect sizes published in other review articles (where available and compatible) were used so that they could be included in the meta-analysis, as referenced in the results section.

### 2.3. Data extraction and analysis

Information regarding study design variables and sample characteristics was extracted for each of the articles in the final selection. The relevant elements of this information were incorporated into a summary of methodological quality and risk of bias across studies. Separate meta-analyses were conducted for studies grouped according to whether symptoms of mental disorder present in their samples were related to psychosis, in order that heterogeneity between studies within each meta-analysis was kept to a level that permitted meaningful comparison. It was assumed that samples from different studies were independent where no evidence existed to suggest otherwise. For studies that measured metacognition across multiple groups with different symptom levels, the effect size calculated was based on the difference between the group with the lowest symptoms of psychopathology (usually the “healthy control” group) and the group with the highest. Where metacognition was measured in the same sample across different cognitive domains, data from the perceptual domain was entered into the meta-analysis, and if more than on perceptual domain was investigated, the visual perception data was included, since first order performance is easier to control in the perceptual domain and visual tasks have been the common method of experimental investigation of metacognition. Analysis of pre-calculated effect sizes was conducted in the IBM SPSS Statistics (Version 28.0, IBM Corp, 2021) using the continuous meta-analysis procedure with DerSimonian and Laird (1986) random-effects estimation including Hartung and Knapp (2001) adjustment to reflect uncertainty in between-study heterogeneity estimation as recommended by Deeks et al. (2019). This analysis produced statistics for heterogeneity of effect sizes, a summary effect size, moderator effects and publication bias for both groups of studies.

#### 2.3.1. Individual effect sizes

Individual effect sizes were estimated for each study by calculating Hedges’ g, an effect size of standardized mean difference, produced by dividing the difference in group means by a value for standard deviation pooled across study groups, which is adjusted for bias arising from small sample size (Hedges and Olkin, 1985; Wilson, 2021). The studies’ effect sizes, with corresponding standard error (SE) were computed using the web-based effect-size calculator developed by Wilson (2001).

#### 2.3.2. Heterogeneity

The presence of significant heterogeneity across study effect sizes was investigated by calculating Cochran’s Q statistic as well as the I^2^ statistic to reflect the percentage of the total variation due to between-study variability, as recommended by Higgins and Thompson (2002).

#### 2.3.3. Summary effect size

To produce the summary effect size for each meta-analysis, the DerSimonian and Laird (1986) random-effects model was applied, weighting individual effect sizes by a combination of within and between-study variance to produce a summary effect size.

#### 2.3.4. Moderator analysis

Given previous findings that metacognition is domain-specific (e.g., Fleming et al., 2014), a Q statistic was calculated as a test for homogeneity of studies grouped by cognitive domain of first-order task, to assess whether the cognitive task used in studies was linked to significant variability in effect sizes.

#### 2.3.5. Publication bias

Risk of publication bias was assessed by producing a funnel plot of study effect sizes relative to their standard error, accompanied by calculation of Egger’s regression test (Egger et al., 1997) of funnel plot asymmetry.

## 3. Results

### 3.1. Systematic review of literature search results

**FIGURE 2:**
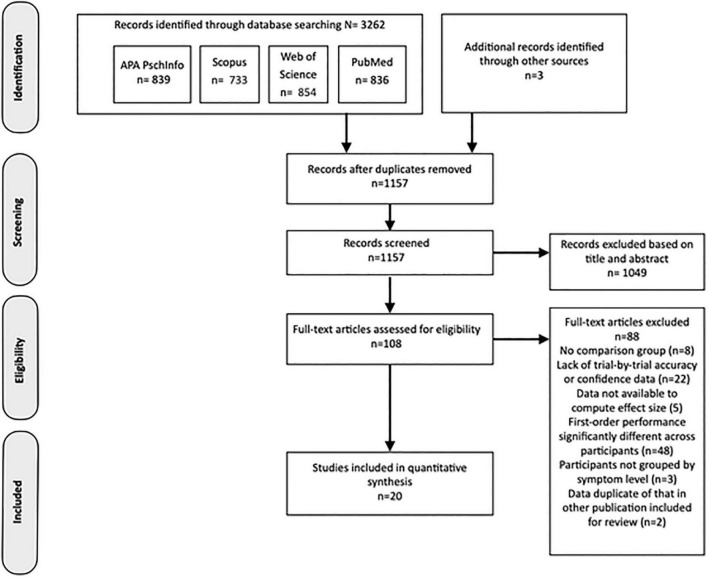
PRISMA flowchart representing the process producing the final selection of studies included in the review.

The results of the study identification, screening and selection process are outlined in the PRIMSA diagram in Figure 2. At the end of this process, 20 studies from the literature search were determined eligible for inclusion in the review.

### 3.2. Study characteristics

The symptoms of mental disorder measured across the 20 studies from the literature search which met the eligibility criteria were more frequently linked to psychosis (*n* = 12), while studies measuring non-psychotic mental disorder (*n* = 8) included samples with symptoms of compulsivity (*n* = 4), addictive disorder (*n* = 3) and functional cognitive disorder (*n* = 1). Across studies of various symptom profiles, the majority used samples whose symptoms were present at a level warranting clinical diagnosis (*n* = 17). The three non-clinical studies used self-report questionnaire scores to group participants according to relative levels of psychopathology symptoms. Studies that used perceptual first-order tasks to assess metacognition (*n* = 12) equated first-order performance between groups and those that used non-perceptual tasks (*n* = 8) found performance to be non-significantly different without manipulation. Perceptual tasks were most frequently in the visual modality (*n* = 11), with only one study using an auditory task (Powers et al., 2017). Of the non-perceptual tasks employed, most assessed memory (*n* = 5), one involved general knowledge, one emotion discrimination and another response inhibition. Study design variables and sample sizes for the studies included in the review are presented in Table 4.

**TABLE 4:** Characteristics of studies included in the review: ‘Psuchop. Participants’ refers to psychopathological participants.

References	Psychop. participants	Control participants	Measure of symptoms	First-order task	Performance comparison	Measure of metacognition
Ben Shachar et al. (2013)	25	22	Obsessive- compulsive tendencies measured by OCI-R	General knowledge: 2 alternative forced-choice recognition	No difference	Goodman– Kruskall gamma coefficient
Berna et al. (2019)	10	9	DSM 4 schizophrenia diagnosis	Memory: Autobiographical recognition	No difference	Meta-d’-d’
Bhome et al. (2022)	14	42	FCD diagnosis through specialist neuropsychiatry service	Perceptual: Visual discrimination	Equated	Meta-d’/d’*
Chan et al. (2015)	22	20	Schizotypal traits as measured by SPQ-BR	Response inhibition: Flanker task	No difference	Low confident incorrect responses
Davies et al. (2018)	31	18	DSM 4 FEP diagnosis	Perceptual: Visual discrimination	Equated	Meta-d’/d’
Eisenacher et al. (2015)	21	38	FEP according to DSM 4 criteria	Memory: Word recognition	No difference	Confidence gap**
Faivre et al. (2021)	21	20	DSM 5 schizophrenia spectrum diagnosis	Perceptual: Visual discrimination	Equated	Meta-d’/d’*
Gaweda et al. (2018)	25	33	DSM 4 FEP diagnosis	Memory: Recognition of prior actions	No difference	Knowledge Corruption Index**
Hauser et al. (2017)	20	20	Trait compulsivity as measured by PI-WSUR	Perceptual: Visual discrimination Perceptual:	Equated	Meta-d’/d’*
Hoven et al. (2022)	27	55	Clinical assessment for gambling disorder	Visual discrimination	Equated	Meta-d’/d’*
Hoven et al. (2022)	28	55	Clinical assessment for OCD	Perceptual: Visual discrimination	Equated	Meta-d’/d’*
Jia et al. (2020)	38	38	Clinical assessment through positive and negative symptoms scores (PANSS)	Perceptual: Visual discrimination	Equated	AUROC2
Kircher et al. (2007)	27	19	DSM 4 FEP diagnosis	Memory: Word recognition	No difference	Relationship between confidence and error responses
Koizumi et al. (2020)	17	18	DSM 4 schizophrenia diagnosis.	Perceptual: Visual detection	Equated	Meta-d’/d’
Moeller et al. (2016)	8	13	DSM 4 SUD diagnosis	Perceptual: Visual discrimination	Equated	Meta-d’/d’
Moritz et al. (2012)	23	29	DSM 4 schizophrenia diagnosis	Emotion discrimination	No difference	High confident incorrect responses
Powers et al. (2017)	15	15	DSM 4 schizophrenia diagnosis	Perceptual: Auditory detection	Equated	Meta-d’/d’*
Sadeghi et al. (2017)	23	24	DSM 4 SUD	Perceptual: Visual	Equated	Meta-d’/d’
Tekcan et al. (2007)	25	27	DSM 4 OCD diagnosis	Memory: Semantic recognition	No difference	Goodman– Kruskall gamma coefficient
Wright et al. (2020)	48	68	ICD 10 FEP diagnosis	Perceptual: Visual	Equated	Meta-d’/d’

First-order performance denoted as “equated” indicates that accuracy was manipulated to be equal between participants, whereas “no difference” indicates that accuracy was not significantly different between participants in the absence of specific experimental manipulation to produce this result. OCI-R, obsessive compulsive inventory - Revised; DSM, Diagnostic and Statistical Manual of Mental Disorders; SPQ-BR, Schizotypal Personality Questionnaire-Brief Revised; FEP, first episode psychosis; PI-WSUR, Padua Inventory-Washington State University Revision; PANSS, The Positive and Negative Syndrome Scale; OCD, obsessive-compulsive disorder; SUD, substance use disorder; ICD, International Statistical Classification of Diseases and Related Health Problems. Descriptions of the metacognitive measures can be found in Table 1. *Studies used hierarchical Bayesian modeling to generate group-level parameter estimates **Studies whose effect sizes are the values cited in Rouy et al. (2021).

### 3.3. Assessment of methodological quality and risk of bias

It has been demonstrated that measures of metacognitive sensitivity are compromised in validity if they can be influenced either by general confidence bias or first-order performance (Galvin et al., 2003). The unwanted influence of first-order performance has in some cases been addressed by equating this across participants, although this has been argued to inflate estimates of metacognitive ability (Rahnev and Fleming, 2019). The twelve studies included in the review employing tasks in the perceptual domain used performance-equating techniques, usually staircase-adjustment procedures, in order to make first-order performance comparable between participants. For the studies using non-perceptual tasks, which neither implemented such a procedure nor used model-based metrics that take into account type-1 d’, the assumption that metacognitive sensitivity measures are not confounded by first-order accuracy relied on follow-up analysis demonstrating nonsignificant performance difference between groups. In these instances, the degree of performance accuracy potentially varies more between the groups of participants and so may produce systematic differences in estimates of metacognitive sensitivity compared to studies equating performance. Where authors did not report results using the meta-d’/d’ or meta-d’-d’ metrics, which was the case for eight of the included studies, insufficient data was available to enable the computation of these measures of sensitivity relative to first-order performance in studies.

### 3.4. Metacognitive sensitivity in the presence of psychosis-related symptoms

#### 3.4.1. Individual and summary effect sizes

**FIGURE 3:**
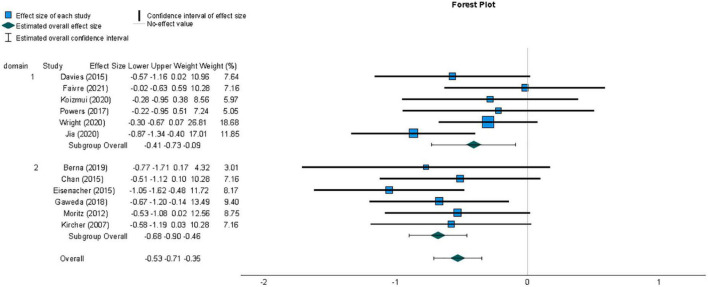
Forest plot of the distribution of Hedges’ g effect sizes for metacognitive sensitivity across studies of samples with psychosis-related symptoms, based on a random- effects analysis, displaying effects by arranged sub-group of task domain, which was either perceptual (1) or non-perceptual (2). Lower metacognitive sensitivity in those with psychosis-related symptoms is indicated by a negative effect size. The summary effect size is indicated by a diamond marker, underneath the individual study effect sizes.

A meta-analysis was conducted to compare effect sizes for differences in metacognitive sensitivity between samples with and without psychosis-related symptoms, using the Hedges’ g form of standardized mean difference as an estimate of effect size. The individual effect sizes, their 95% Confidence Interval (CI) and the weight with which they contribute to the summary effect size are represented in the forest plot in Figure 3. The summary effect size can be considered to be of medium magnitude based on guidelines recommended for a related measure of standardized mean difference, Cohen’s d (Cohen, 1988), and indicated lower metacognitive sensitivity in those with psychosis-related symptoms compared to those not displaying psychopathology (*g* = −0.53, 95% CI = −0.71, −0.35). A t-test indicated that the observed summary effect was very unlikely to have arisen by chance (*t*(11) = −6.34, *p* < 0.001).

#### 3.4.2. Heterogeneity

The existence of heterogeneity between study effect sizes was tested by calculating the Q statistic, which failed to indicate significant heterogeneity (Q = 11.16, df = 11, *p* = 0.43). The proportion of variability between effect sizes not attributable to sampling error was quantified using the I^2^ statistic (Higgins and Thompson, 2002) and this was found to be negligible ( < 1%).

#### 3.4.3. Moderator analysis

Although there was no evidence for heterogeneity across studies included in the analysis, a planned investigation was conducted as to whether variation in effect sizes was related to the cognitive domain of the first-order task used, given previous findings that metacognition is domain-specific (e.g., Fleming et al., 2014). The Q statistic calculated as a test for homogeneity of studies grouped by cognitive domain of first-order task did not provide evidence of a moderating effect of cognitive domain task on measures of metacognitive sensitivity (*Q* = 2.32, df = 1, *p* = 0.13).

#### 3.4.4. Publication bias

**FIGURE 4:**
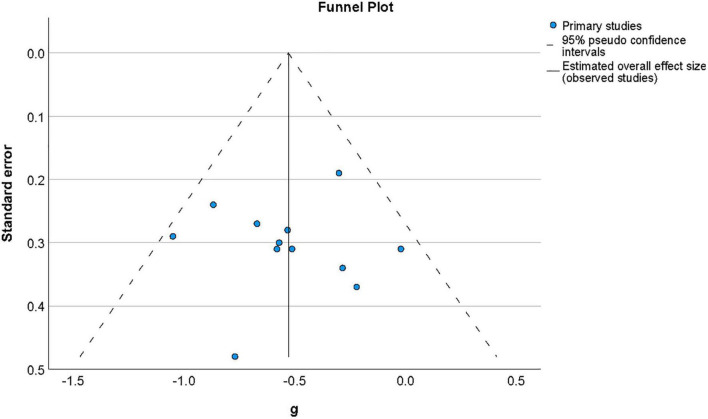
Funnel plot of the distribution of effect sizes by their standard error for studies of metacognition in the presence of psychosis-related symptoms. The vertical line indicates the value of the summary effect size. The area of the graph within the triangle represents the values which samples have 95% probability of showing if variance is homogeneous. Funnel plot of the distribution of effect sizes by their standard error for studies of metacognition in the presence of psychosis-related symptoms.

Publication bias was examined by assessing the symmetry of the distribution of included effect sizes in terms of their precision. Figure 4 shows a funnel plot constructed around the summary effect size and represents the area within which 95% of studies should fall in the absence of heterogeneity and biases (Deeks et al., 2019). The lack of marked asymmetry in the funnel plot as depicted in Figure 4 suggested there is no significant publication bias for results in this area, which was supported by a non-significant value for Egger’s regression test (Egger et al., 1997) of funnel plot asymmetry (*t*(11) = −0.77, *p* = 0.94).

### 3.5. Metacognitive sensitivity in the presence of non-psychotic symptoms of mental disorder

#### 3.5.1. Individual and summary effect sizes

**FIGURE 5:**
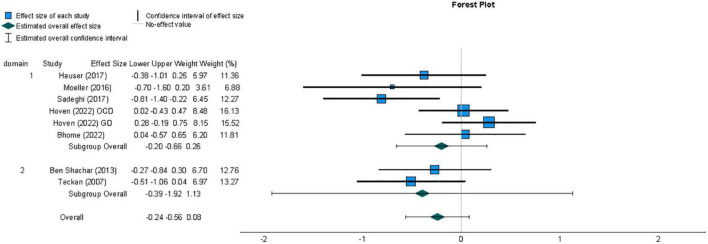
Forest plot of the distribution of effect sizes for metacognitive sensitivity across studies of samples with non-psychotic symptoms of mental disorder, based on a random-effects analysis, displaying effects by arranged sub-group of task domain, which was either perceptual (1) or non-perceptual (2). Lower metacognitive sensitivity in those with non-psychotic symptoms of mental disorder is indicated by a negative effect size. The summary effect size is indicated by a diamond marker, underneath the individual study effect sizes.

A second meta-analysis was conducted for effect sizes across studies comparing metacognitive sensitivity in samples with non-psychosis-related symptoms of mental disorder and healthy individuals. The resulting summary effect size, reflecting the weighted averages of individual studies’ effect sizes, is displayed in Figure 5. Unlike the meta-analysis for studies investigating metacognition in individuals with psychosis-related symptoms, the summary effect size for this group of studies did not provide evidence of an overall difference in metacognition compared to those without symptoms of mental disorder (*g* = −0.24, 95% CI = −0.56, 0.08). A *t*-test indicated that the observed summary effect was not significant t(7) = −1.78, *p* = 0.12).

#### 3.5.2. Heterogeneity

The existence of heterogeneity between study effect sizes was tested by calculating the Q statistic, which failed to indicate significant heterogeneity (*Q* = 12.43, df = 7 *p* = 0.09). The proportion of variability between effect sizes not attributable to sampling error was quantified using the I^2^ statistic (Higgins and Thompson, 2002); this did indicate that a moderate degree of heterogeneity existed between studies (I^2^ = 44%; Deeks et al., 2019).

### 3.5.3. Publication bias

**FIGURE 6:**
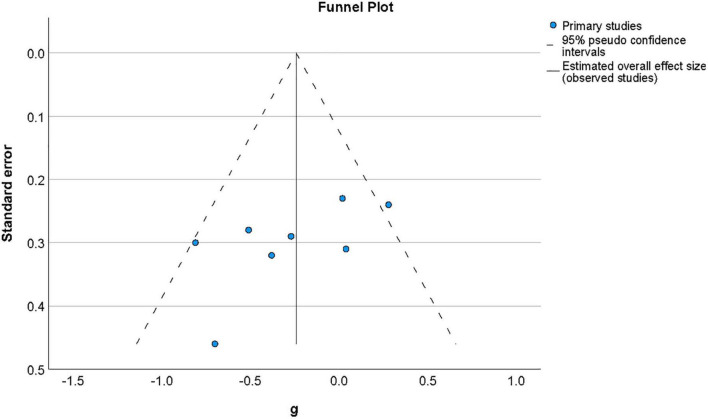
Funnel plot of effect sizes by standard error for studies of metacognition in the presence of non-psychotic symptoms of mental disorder. The vertical line indicates the value of the summary effect size. The area of the graph within the triangle represents the values which samples have 95% probability of showing if variance is homogeneous.

Publication bias was by examined by assessing the symmetry of the distribution of included effect sizes in terms of their precision. Figure 6 shows a funnel plot constructed around the summary effect size and represents the area within which 95% of studies should fall in the absence of heterogeneity and biases (Deeks et al., 2019). The asymmetry of distribution in the funnel plot as depicted in Figure 6 suggested there may be a publication bias for results in this area, although a non-significant value was found for Egger’s regression test (Egger et al., 1997) of funnel plot asymmetry (t(7) = −1.88, *p* = 0.11). It has, however, been argued that both funnel plot symmetry may fail to accurately reflect publication bias where the number of studies included is low (Begg and Mazumdar, 1994), as it the case for this meta-analysis.

#### 3.5.4. Moderator analysis

As for studies of psychosis-related symptoms, a planned investigation was conducted as to whether effect sizes varied for studies measuring metacognition using tasks from perceptual or non-perceptual cognitive domains. Although there was no evidence for heterogeneity across studies included in the analysis, a planned investigation was conducted as to whether variation in effect sizes was related to the cognitive domain of the first-order task used, given previous findings that metacognition is domain-specific (eg., Fleming et al., 2014). As with the previous meta-analysis, the Q statistic calculated as a test for homogeneity of studies grouped by cognitive domain of first-order task did not provide evidence of a moderating effect of cognitive domain task on measures of metacognitive sensitivity (*Q* = 0.52, df = 1, *p* = 0.47).

## 4. Discussion

### 4.1. Summary

This systematic review and meta-analysis aimed to establish whether metacognitive sensitivity differs between those with and without symptoms of mental disorder. Metacognitive sensitivity was defined as the ability to discriminate first-order response accuracy through reports of confidence with respect to individual responses in a task. We further sought to test whether this depended on the domain of the first-order cognitive task used to measure metacognitive sensitivity, in particular whether there were any differences between studies employing perceptual versus non-perceptual tasks. The results showed that metacognitive sensitivity was significantly reduced in those with symptoms of mental disorder related to psychosis but only a (nonsignificant) trend could be observed in those with symptoms related to obsessive-compulsive disorder (OCD), substance use disorder (SUD) or functional cognitive disorder (FCD). The effects found are based on studies using tasks requiring first-order cognition in different domains, here categorized as perceptual versus non-perceptual. No evidence was found that the cognitive domain moderated metacognitive sensitivity, since the between-group Q statistic for studies grouped as perceptual and non-perceptual did not reach significance in either meta-analysis.

### 4.2. Relation of findings to existing literature

The results of the meta-analyses in this review converge to some extent with the conclusions drawn by Hoven et al. (2019) from a qualitative review of research into metacognitive bias and sensitivity in populations with diverse symptoms of mental disorder, also conducted across a variety of first-order cognitive domains. Hoven et al. found that the balance of evidence suggested impairment of metacognitive sensitivity in those with subclinical obsessive-compulsive tendencies as well as in those with psychosis-related symptoms. However, research relating to symptoms of clinical OCD, addiction, anxiety and depression was either absent or provided mixed evidence for impairment of metacognitive sensitivity. The difficulty in drawing conclusions from many of the studies about metacognitive sensitivity in the review by Hoven et al. is due to the possibility that they were confounded by confidence biases and first order performance. The studies in the current review have equated or not significantly different first-order performance, reducing the possibility of bias in estimating metacognitive sensitivity, although studies employing non-perceptual tasks have measures with higher risk of being influenced by first-order performance.

The research of Rouault et al. (2018b) specifically related variability in metacognitive efficiency, a measure of metacognitive sensitivity relative to performance, across symptoms ascribed to different diagnostic categories, which has parallels with the findings of the current review. Rouault, Seow et al. found that metacognition was predicted in a general population sample by transdiagnostic symptom dimensions derived through factor analysis of individual items from a range of psychiatric questionnaires, replicating a latent structure across diagnostic categories originally obtained by Gillan et al. (2016). Metacognitive efficiency was found to be negatively correlated with scores in one kind of symptom dimension, characterized by compulsivity and intrusive thoughts, while it was positively correlated with scores on another dimension, characterized by anxiety and depression. While the indication of a negative association between metacognitive efficiency and the compulsivity-intrusive thought dimension is in agreement with the direction of the trend found in the current meta-analysis of studies involving compulsivity and addiction, although the overall effect did not reach significance. However, no research involving symptoms of anxiety or depression met criteria for inclusion in the current review. Based on the research by Rouault, Seow, et al. and other findings that fail to demonstrate any impairment in sensitivity in relation to symptoms of depression (Herskovik et al., 1986; Fieker et al., 2016; Moses-Payne et al., 2019), it seems unlikely that the effect found for those symptoms of psychopathology included in this analysis can be generalized to symptoms of anxiety or depression.

The results in the current review follow the direction of findings in another recent meta-analysis by Rouy et al. (2021) synthesizing research in samples with diagnoses of schizophrenia spectrum disorders, which found a strong overall effect size (*g* = −0.57) for reduced metacognitive sensitivity in those with schizophrenia compared to control participants. This effect size was however based on a selection of studies that did not universally control for first-order performance. Rouy et al. performed further analyses which indicated that the magnitude of the summary effect size was substantially reduced when estimated only from those studies actively matching first-order performance across participants. The reduced effect size found by Rouy et al. (2021) is at odds with the larger effect found for symptoms of psychosis in the current review, which also included only studies that equated first-order performance, or which demonstrated no significant difference between groups’ performance. The larger effect size for studies of psychosis-related symptoms in the current analysis may be attributable to the inclusion of those studies in which performance was not actively equated, only not significantly different.

### 4.3. Associations of metacognition with neuroanatomical and functional activation differences

Attempts to explain interindividual differences in metacognition have assessed metacognitive sensitivity in relation to neuroanatomical features and task-related activation. Fleming et al. (2010) found that strategic metacognition was predicted by individual differences in the structure of anterior prefrontal cortex, while the dorsolateral prefrontal cortex has been causally implicated in metacognition accuracy through application of repetitive transcranial magnetic stimulation (Rounis et al., 2010). Since structural and functional atypicality in areas of the prefrontal cortex have been linked to a range of mental disorders (Koenigs and Grafman, 2009; Goldstein and Volkow, 2011), the evidence for the role of prefrontal areas in metacognitive processing contributes to an expectation of impaired metacognitive sensitivity across different kinds of mental disorder. This neuroanatomical link is supported by studies in the current review; Moeller et al. (2016) found that decreased gray matter volume in the anterior prefrontal cortex predicted the degree of perceptual metacognitive impairment in substance use disorder and research by Jia et al. (2020) indicated that differences in schizophrenia patients’ metacognition were linked to frontoparietal hypoactivity. However, the absence of an association between impaired perceptual metacognitive sensitivity in psychosis and gray matter volume reduction in the prefrontal cortex in the study by Davies et al. (2018) means that further research is required in order to clarify the contribution of this region to metacognitive sensitivity. Furthermore, a review of neuroanatomical associations identified in relation to metacognition has suggested that involvement of substrates in metacognition may depend on the cognitive domain in which it is exercised, and that metamemory performance may be more reliant on structures outside of the prefrontal cortex (Vaccaro and Fleming, 2018).

### 4.4. Domain-generality of metacognitive performance

A recent review has investigated whether individuals’ metacognitive performance is correlated across distinct cognitive domains and found that evidence was inconclusive regarding the domain-general nature of metacognition (Rouault et al., 2018a). In the current review, those studies using tasks in the perceptual domain were able to actively equate performance between groups, while studies using non-perceptual tasks were not. This has the implication that any differences in effects between sub-groups distinguished by task domain may reflect not only the influence of domain but also that of equating first-order performance. The lack of significant variability in effect sizes between the sub-group of studies using perceptual tasks and equating performance and the subgroup using non-perceptual tasks without equating performance can be interpreted in two possible ways. Firstly, it is possible that neither task domain nor control of performance significantly influence effect size. While this does not refute that metacognitive capacity may differ across first-order cognitive domains, it would imply that its impairment is observed to the same extent across domains in those with psychiatric symptoms. Secondly, it is possible that different degrees of metacognitive impairment do exist for different first-order domains but that this is offset by the influence of the co-varying performance manipulation. It should also be considered that differences in metacognitive sensitivity between cognitive domains may be obscured by the fact that there is also variation in terms of the stimuli and measures used within domains. Stimulus-level variables have been shown to produce different estimates of metacognition, as in the case of spatial frequency (Koizumi et al., 2020) for perceptual metacognition or episodic rather than semantic content (Tekcan et al., 2007; Berna et al., 2019) for metamemory, while different metrics within domains also reduce the clarity of cross-domain comparisons. The heterogeneous combinations of design and measurement features across studies prevent any clear conclusions regarding the influence of any individual factor on estimates of metacognitive sensitivity.

### 4.5. Interpreting measures of metacognitive performance

Some challenges have been raised with respect to conclusions drawn from the measures of metacognitive sensitivity employed in the studies reviewed, which have implications for the interpretation of the group differences observed. Metacognitive monitoring has been conceived as involving the processing of internal evidence arising from first-order cognition (Fleming et al., 2012) and that equating first-order performance provides that the evidence available for metacognitive processing is also equated, enabling a measure of true metacognitive sensitivity. It has been argued by Paulewicz et al. (2020) that neither equating first-order performance nor implementing model-based measures of second-order sensitivity that are formulated relative to first-order sensitivity are sufficient to isolate metacognitive performance from first-order processes. Paulewicz et al. (2020) raise this concern in the context of a causal analysis framework, taking into account that metacognition may involve the monitoring or regulation of multiple interacting stages which constitute first-order processing for a given task (Flavell, 1979). The authors argue that equalizing first-order accuracy may not standardize all first-order processes or metacognitive regulatory activity, which means that intended measures of metacognitive sensitivity may still reflect differences in first-order processes or metacognitive regulation where first-order performance is the same. Following this reasoning, the effect for differences in metacognitive measures between groups may require a more limited interpretation as a difference in the statistical relation between accuracy and confidence, rather than differential operation of a metacognitive monitoring process.

### 4.6. Correspondence of local metacognition to global metacognition and mental health

Although it is difficult to specifically isolate metacognitive monitoring through empirical measures, it is useful to compare the relation of second-order judgments to standardized behavioral measures of cognition. This operationalization of metacognition, based on relatively discrete cognitive functions involved in task performance, allows more direct interindividual comparison of second-order judgments than attributive measures of metacognition, which are based on synthesizing diverse sets of cognitive processes across contexts (Lysaker et al., 2018). Nevertheless, the complexity of cognitive processes varies even across task-based metacognition, given that some studies involve tasks which require only low-level stimulus processing in terms of contrast or global motion (Fleming et al., 2010; Hauser et al., 2017) whereas others involve of stimuli like facial expressions which are more likely to invoke multiple or high-order cognitive processes (Moritz et al., 2012). These latter examples may assess cognitive processes more comparable to those represented by measures of global metacognition such as the Metacognitions Questionnaire (MCQ; Cartwright-Hatton and Wells, 1997). The correspondence between local and global forms of metacognition and their contribution to mental health is receiving increased attention in recent research (for a review see Seow et al., 2021). Although Lee et al. (2021) found that global metacognition is based on integrating instances of local metacognition, the information was not optimally integrated and influenced by additional variables. Chan et al. (2015) compared both local and global metacognition in individuals with high and low schizotypy. Chan et al. (2015) found that groups differed significantly only in measures of global metacognition as reported in the MCQ, not local metacognition. More recently, Bhome et al. (2022) also found evidence of a dissociation between local and global metacognition, reporting intact local metacognition in FCD patients but reduced global metacognition. Further comparison of local and global metacognition would help to clarify the extent to which these are differentially impacted in psychopathology.

To determine the functional relevance of task-based measures of metacognitive ability to the assessment or treatment of those demonstrating symptoms of psychopathology it is important to consider whether these measures specifically predict symptom severity or broader functioning. Where significant overall reductions in metacognitive sensitivity have been found for samples with psychiatric symptoms relative to control groups, metacognitive performance has predicted symptom severity in some cases (Eisenacher et al., 2015; Jia et al., 2020) but not others (Kircher et al., 2007; Davies et al., 2018; Gaweda et al., 2018). As the current review included only three studies for sub-clinical level psychiatric symptoms, it was not viable to use sub-group analysis to evaluate whether the extent of metacognitive impairment depended on symptom severity reaching a clinical threshold. Where metacognition is assessed in relation to symptoms that present at a prodromal level or within an initial episode of psychosis, the extent of metacognitive impairment may guide clinical intervention if this is able to predict likelihood of future episodes or recovery prognosis (Hauser et al., 2017). At present, no study fulfilling our criteria has implemented a longitudinal design to establish the potential of metacognitive assessment in this respect.

### 4.7. Limitations of the current review

The current review’s conclusions regarding metacognitive impairment in psychopathology may be considered limited in the sense that they only reflect research involving between-group comparisons, so overlook findings relating continuous measures of psychiatric symptoms to metacognitive sensitivity (Rouault et al., 2018b; Moses-Payne et al., 2019). Conclusions of impaired metacognitive sensitivity are based only on groups with symptoms of psychosis, compulsivity, addiction or FCD as only these met pre-defined eligibility criteria regarding experimental design. For this reason, the results of analyses in this review are not informative with respect to other kinds of psychiatric symptoms. A factor that limits the conclusions that can be drawn regarding effects for the specific disorders in the reviewed studies is that only measures of the primary symptom(s) of interest characterizing the groups are reported, with a few exceptions (Eisenacher et al., 2015; Faivre et al., 2021). Given the presence of co-morbidity among many psychiatric diagnoses, and growing evidence for the existence transdiagnostic symptom clusters (Rouault et al., 2018b), it is appropriate to assess the potential influence on outcome measures of other symptoms beyond those primarily characterizing a sample. This is particularly relevant for symptoms of depression, which may vary considerably between participants with a similar psychiatric diagnosis as well as between participants assigned to an asymptomatic or low-symptom control group (Rouy et al., 2021). Where studies do not determine the presence of secondary symptoms or assess the relation of these to the outcome measures, their contribution to metacognitive differences attributed to the primary disorder cannot be evaluated.

Potential sources of bias in the studies included for review relate to the use of outcome measures that are vulnerable to first-order performance and confidence bias confounds (Fleming and Lau, 2014). A risk of confidence bias impacting measures of metacognitive sensitivity is found in those studies in non-perceptual domains comparing group sensitivity with measures based on the absolute number of incorrect responses given with high or low confidence or confidence-accuracy correlation (Masson and Rotello, 2009), a consideration particularly relevant to the current review insofar as this has been found to be heightened in some psychiatric populations (Hoven et al., 2019). A clearer understanding of the relationship between psychiatric symptoms and metacognitive sensitivity could be reached if future studies increase use of measures such meta-d’/ d’ which are not influenced by response bias and standardized in terms of first-order performance, allowing findings to be more easily related across samples and task procedures reliant on cognition in different domains (Fleming, 2017). Finally, it is important to note that since the studies included for review were all cross-sectional in nature, it is not possible to arrive at conclusions regarding the causal role of metacognitive impairments in producing associated symptoms.

## 5. Conclusion

This systematic review and meta-analysis of findings from research into metacognitive monitoring in individuals with psychiatric symptoms has provided evidence that metacognitive sensitivity is reduced in populations with symptoms related to psychosis. The overall effect found for dysfunction of metacognitive monitoring in those demonstrating features of psychotic psychopathology suggests that this, alongside particular impairments in first-order processes depending on the symptom profile (Hauser et al., 2017; Gaweda et al., 2018), may be implicated in the manifestation of these symptoms. There was however no conclusive evidence of reduced metacognitive sensitivity in those with non-psychotic symptomatology. No evidence was found to suggest second-order performance was dependent on specific to first-order cognitive domain in either meta-analysis. Future research using longitudinal designs may clarify the role of metacognitive sensitivity in the prognosis of psychiatric symptoms and mental disorder, which can guide decisions to recommend interventions of the kind which have been found to improve metacognitive sensitivity in healthy individuals (Baird et al., 2014) for those with psychiatric symptoms. A further avenue of research to clarify the functional relevance of metacognitive impairment could investigate how metacognitive sensitivity affects consequent cognition and behavior, which has as yet received limited attention (Ben Shachar et al., 2013) and which may also clarify how local and global forms of metacognition are related (Chan et al., 2015).

## Data availability statement

The original contributions presented in this study are included in this article/supplementary material, further inquiries can be directed to the corresponding authors.

## Author contributions

All authors listed have made a substantial, direct, and intellectual contribution to the work, and approved it for publication.
